# Widely Targeted Metabolomics Decodes Metabolic Remodeling and Functional Shifts in *Ganoderma lucidum*-Fermented Green Tea Infusion

**DOI:** 10.3390/foods14162855

**Published:** 2025-08-18

**Authors:** Xuzhou Liu, Ying Ju, Shuai Wen, Hongzhe Zeng, Chao Wang, Mingguo Jiang, Bingchuan Tian, Jianan Huang, Zhonghua Liu

**Affiliations:** 1 Key Laboratory of Tea Science of Ministry of Education, National Research Center of Engineering and Technology for Utilization of Botanical Functional Ingredients, Co-Innovation Center of Education Ministry for Utilization of Botanical Functional Ingredients, Key Laboratory for Evaluation and Utilization of Gene Resources of Horticultural Crops, Ministry of Agriculture and Rural Affairs of China, Hunan Agricultural University, Changsha 410128, China; wyu2017ws@163.com (S.W.); zenghongzhe@hotmail.com (H.Z.); wangchao2809@126.com (C.W.); jian7513@hunau.edu.cn (J.H.); 2 Institute of Microbiology, Guangxi Crop Genetic Improvement and Biotechnology Laboratory, Guangxi Academy of Agricultural Sciences, Nanning 530007, China; 13578935348@163.com; 3 Higentec Limited Company, Changsha 410125, China; tianbc@higentec.com; 4 Guangxi Key Laboratory for Polysaccharide Materials and Modifications, School of Marine Sciences and Biotechnology, Guangxi Minzu University, Nanning 530006, China; mzxyjiang@gxun.edu.cn

**Keywords:** fermented tea, *Ganoderma lucidum*, widely targeted metabolomics, lipid-lowering activity

## Abstract

This study used a targeted metabolomics approach to examine changes in metabolites within green tea infusions fermented by *G. lucidum* (TFG) and evaluate the in vitro antioxidant and lipid-lowering properties of TFG. Fermentation decreased tea polyphenols, flavonoids, caffeine, soluble sugars, theaflavins, and catechins, while increasing free amino acids and theabrownins. The microbial bioconversion process led to the generation of decorated flavonoids, phenolic acids, terpenoids, alkaloids, nucleotides, and amino acids. This process shifted the tea’s taste from bitter and astringent to mellow, primarily due to the transformation of flavonoid glycosides, caffeine, catechins, 5′-guanosine monophosphate, 5′-uridine monophosphate, and theabrownins. Volatile metabolites added woody, floral, sweet, and fruity aromas. Reduced gallic acid and catechins lowered antioxidant activity, whereas increased theabrownins enhanced lipid-lowering activity and imparted a reddish-brown color. These findings indicate that fermentation significantly affects the flavor, aroma, and lipid-lowering ability of green tea infusion.

## 1. Introduction

Microbial fermentation enhances green tea’s value by improving its aroma, flavor, and bioactive compounds, making it more appealing to consumers. Fermentation with *Saccharomyces boulardii*, *Lactiplantibacillus plantarum*, and *Mycetinis scorodonius* produces unique fruity, minty, and chocolate-like flavors [1,2]. *Aspergillus niger* reduces astringency and increases theabrownins, which offer health benefits [3]. Two-step fermentation with lactic and acetic acid bacteria boosts bioactive compounds like γ-aminobutyric acid and lactic acid, enhancing antioxidant effects [4]. Fermented teas, such as *Monascus purpureus*-fermented pu-erh tea, are more effective in lowering lipids and inhibiting inflammation than unfermented teas due to the transformation of functional ingredients [5]. Compounds like theaflavins and theasinensins formed during fermentation may reduce body weight and triglycerides, offering anti-obesity and hypotriglyceridemic benefits [6]. Thus, microbial fermentation improves both the sensory and health aspects of green tea.

*Ganoderma lucidum*, a basidiomycete, is a renowned edible and medicinal macrofungus known for its bioactive compounds and diverse pharmacological effects [7]. It contains triterpenoids, polysaccharides, sterols, steroids, and nucleotides. Studies have confirmed its pharmacological effects, including antioxidant [8], immunomodulatory [9], anti-diabetic [10], anti-inflammatory [11], anti-tumor [12], and neuroprotective [8] properties.

This study investigates the biomolecular alterations in green tea infusion fermented by *G. lucidum* (TFG), a promising functional food product. The integration of *G. lucidum*—a medicinal fungus with established bioactive compounds and pharmacological properties—into tea fermentation represents a novel strategy to enhance the nutritional and sensory profiles of tea products for the food market. However, the dynamic metabolic changes during this process remain poorly understood. To address this gap, we employed widely targeted metabolomics using UPLC–MS/MS and GC–MS/MS to comprehensively profile non-volatile and volatile metabolites, alongside evaluating antioxidant activity and lipid-lowering potential. These methods, previously applied to tea processing studies [13,14], are here adapted to decode the metabolic remodeling and functional shifts induced by *G. lucidum* fermentation. This work provides critical insights into how fungal fermentation modifies tea constituents, offering a scientific basis for optimizing the development of health-promoting fermented tea products.

## 2. Materials and Methods

### 2.1. Chemicals and Reagents

Caffeine (≥98%) was purchased from Enzo Life Sciences Inc. (Farmingdale, NY, USA). Gallic acid (GA, ≥98%), (−)-epicatechin (EC, ≥99%), (−)-epigallocatechin (EGC, ≥95%), (−)-epicatechin gallate (ECG, ≥98%), (−)-epigallocatechin gallate (EGCG, ≥97%), catechin (C, ≥98%), catechin gallate (CG, ≥98%), (−)-gallocatechin (GC, ≥98%), gallocatechin gallate (GCG, ≥98%), theanine (≥98%), and γ-aminobutyric acid (GABA, ≥99%) were procured from Sigma-Aldrich Co. (St. Louis, MO, USA). Standard amino acids (≥98%), including lysine, histidine, arginine, glutamine, glycine, tryptophan, glutamic acid, leucine, isoleucine, proline, aspartic acid, serine, threonine, asparagine, alanine, tyrosine, phenylalanine, and valine were obtained from ChemFaces (Wuhan, China). Sodium glycinate (≥98%) and sodium taurocholate (≥95%) were obtained from Macklin Co. (Shanghai, China) and Solarbio Co. (Shanghai, China), respectively.

### 2.2. Green Tea Fermentation and Major Chemical Composition Determination

*G. lucidum* was provided by Dr. Wang Xiaoguo from the Guangxi Academy of Agricultural Sciences, China. To prepare the *G. lucidum* inoculum, stored mycelium was reactivated on potato dextrose agar at 28 °C for 1 week. Green tea, purchased from Chenyifan Company (Qingdao, China), was powdered and sieved through a 100-mesh sieve. Twenty grams of tea powder was mixed with 400 mL distilled water in a 1 L flask and sterilized at 121 °C for 20 min. After cooling, the medium was inoculated with the entire mycelial biomass scraped from a fully grown *G. lucidum* slant culture (approximately 100 mg) under aseptic conditions. Fermentation occurred for 12 days at 28 °C, 180 rpm. Samples were taken on days 0, 4, 8, and 12. Green tea infusions were categorized as 0-day (control, CK; control group), 4-day (Treat-d4), 8-day (Treat-d8), and 12-day (Treat-d12, TFG) groups. The supernatant from centrifugation at 4500 rpm, 4 °C, was labeled and stored in liquid nitrogen.

The analysis of catechin, caffeine, and theaflavin is performed utilizing a liquid chromatograph (VanquishCore, Thermo Fisher Scientific, Waltham, MA, USA) in accordance with GB/T 30483-2013 [15]. Meanwhile, the concentrations of tea polyphenol, theabrownin, free amino acid, soluble sugar, and total flavonoids are determined using a UV–visible spectrophotometer (8453, Agilent, Palo Alto, CA, USA) following the methodologies outlined in GB/T 8313-2018 [16], NY/T 3675-2020 [17], GB/T 8314-2013 [18], NY/T 2742-2015 [19], and SN/T 4592-2016 [20], respectively.

### 2.3. Determination of Non-Volatile Metabolites During the Fermentation Process of TFG

#### 2.3.1. Sample Preparation

We used a lyophilizer (Scientz-100F, Scientz, Ningbo, China) to remove moisture from samples via vacuum freeze-drying. The samples were ground into powder with a grinder (Retsch MM 400, Retsch GmbH, Haan, Germany) for 1.5 min at 30 Hz. We then measured 50 mg of sample powder with an electronic balance (MS105DM, Mettler Toledo, Greifensee, Switzerland) and added 1200 μL of pre-cooled (−20 °C) 70% methanolic extract. The mixture was vortexed for 30 sec every 30 min for a total of 6 times. After centrifugation at 12,000 rpm for 3 min, the supernatant was filtered through a 0.22 μm membrane. The filtrate was transferred to an injection vial for UPLC-MS/MS analysis.

#### 2.3.2. UPLC and ESI-Q TRAP-MS/MS Conditions

Sample extracts were analyzed using a UPLC-ESI-MS/MS system (ExionLC™ AD, Framingham, MA, USA) coupled with a tandem mass spectrometry system. The UPLC was equipped with an Agilent SB-C18 column (1.8 µm, 2.1 mm × 100 mm). The mobile phase consisted of solvent A (0.1% formic acid in water) and solvent B (0.1% formic acid in acetonitrile). The gradient program started with 95% A and 5% B, transitioned to 5% A and 95% B over 9 min, held for 1 min, then returned to 95% A over 1.1 min, and held for 2.9 min. The flow rate was 0.35 mL/min, in a column oven at 40 °C, with a 2 μL injection volume. The effluent was connected to an ESI-triple quadrupole-linear ion trap (QTRAP)-MS. The ESI source parameters were ion spray voltage at 5500 V (positive mode) and −4500 V (negative mode), source temperature at 500 °C, GSI at 50 psi, GSII at 60 psi, and curtain gas at 25 psi. Collision-activated dissociation (CAD) was set to high. MRM transitions were monitored with specific DP (declustering potential) and CE (collision energy) settings based on eluted metabolites.

### 2.4. Analysis of the Volatile Metabolites Present in the TFG Samples

#### 2.4.1. Sample Treatment

Samples were weighed, frozen in liquid nitrogen, and stored at −80 °C. Before analysis, they were ground into powder in liquid nitrogen, and 500 mg was transferred to a 20 mL headspace vial (Agilent, Palo Alto, CA, USA). A saturated NaCl solution was added to prevent enzyme activity, and the vial was sealed with crimp-top caps and TFE–silicone septa (Agilent). For SPME analysis, vials were heated at 60 °C for 5 min, followed by a 120 µm DVB/CWR/PDMS fiber (Agilent) introduced into the headspace at 60 °C for 15 min.

#### 2.4.2. GC-MS Conditions

Volatile organic compounds (VOCs) were desorbed from the fiber in the GC system (Agilent 8890) at 250 °C in splitless mode for 5 min. VOCs were identified and quantified using an Agilent 7000E mass spectrometer and a DB-5MS capillary column (30 m × 0.25 mm × 0.25 µm). Helium was used as the carrier gas at 1.2 mL/min. The oven temperature program started at 40 °C for 3.5 min, increased by 10 °C/min to 100 °C, then by 7 °C/min to 180 °C, and finally by 25 °C/min to 280 °C, holding for 5 min. Mass spectra were recorded in electron impact (EI) mode at 70 eV, with the quadrupole, ion source, and transfer line temperatures at 150 °C, 230 °C, and 280 °C, respectively. Analytes were identified using selected ion monitoring (SIM) mode.

### 2.5. Sensory Evaluation, E-Tongue, and E-Nose Analysis

Eight tea experts (four male, four female) conducted a sensory evaluation of liquor color, aroma, and taste following Chinese national standard GB/T 23776-2018 [21]. Briefly, 3.00 g of tea was brewed with 150 mL of boiling water (1:50, *w*:*v*) for 5 min, and the infusion was poured into a bowl for evaluation [22]. Aroma quality was assessed using an E-nose system (FOX 4000, Alpha MOS, Toulouse, France). A 1.000 g tea powder sample was sealed in a 15 mL vial and heated at 60 °C for 20 min, and 2 mL of headspace gas was injected into the E-nose system. Data were recorded for 90 sec using six metal oxide sensors with a 200 sec delay. Air at 0.35 bar and 150 mL/min was the carrier gas [22]. Taste quality was evaluated using an E-tongue (SA402B, Insent, Aichi, Japan) [23]. A 3.00 g sample was brewed with 150 mL of water for 5 min. The E-tongue measured sourness, bitterness, astringency, umami, sweetness, and richness over three phases: sample detection (30 s), aftertaste detection (30 s), and cleaning (120 s). Each sample was prepared in triplicate, and each infusion was measured four times for an average value.

### 2.6. Analysis of Antioxidant Capacity and Lipid-Lowering Activities In Vitro

Antioxidant activities were evaluated using DPPH and ABTS assays, following Afonso et al. [24] with kits from Norminkoda Biotechnology Co., Ltd. (Wuhan, China). Both assays were performed in triplicate for each variety. The bile acid-binding capacity of TFG was assessed using sodium glycinate and sodium taurocholate, based on Dong et al. [25] For simulated digestion, 1 mL pepsin (10 mg/mL) and 1 mL HCl (0.01 mol/L) were added to 1 mL of tea samples (CK, Treat-d4, Treat-d8, Treat-d4, and Treat-d12) and incubated for 1 h. Next, 4 mL trypsin (10 mg/mL) and a salt solution (0.3 mmol/L sodium taurocholate and sodium glycinate in 0.1 mol/L phosphate buffer) were added, followed by incubation at 37 °C for 1 h. The mixture was centrifuged at 9000× *g* for 20 min. The supernatant was mixed with 7.5 mL H_2_SO_4_ (60%) and heated at 70 °C for 20 min, then cooled in an ice bath. Absorbance at 387 nm was measured to calculate the bile acid binding rate using the formula: (A − B)/A × 100, where A is the absorbance of the blank and B is the absorbance of the sample.

### 2.7. Statistical Analysis

Primary metabolomics data were evaluated by comparing fragmentation patterns, retention times, and accurate m/z values with standards in the self-compiled (MetWare, Wuhan, China) and public databases (Metlin, Massbank). Multivariate statistical analyses were systematically performed: principal component analysis (PCA) was conducted using the prcomp function in R with unit variance scaling applied for data normalization; orthogonal partial least squares–discriminant analysis (OPLS-DA) was implemented via the MetaboAnalystR package v4.0.3, where data were log2-transformed and mean-centered prior to model construction, and validated by a 200-permutation test to mitigate overfitting. Differential metabolites were identified by combining variable importance in projection (VIP > 1) and absolute log_2_-fold change values (|Log_2_FC| ≥ 1.0). Hierarchical cluster analysis (HCA) was applied to generate clustered heatmaps with dendrograms using the R package ComplexHeatmap package v2.16.0, in which color gradients represent UV-scaled normalized signal intensities; Pearson correlation coefficients (PCCs) between samples were calculated using the cor function and visualized as correlation heatmaps. Statistical significance was determined using the hypergeometric test at a significance threshold of *p* < 0.05.

## 3. Results and Discussion

### 3.1. Changes in Major Chemical Components

As shown in Table 1, fermentation resulted in a noticeable decline in tea polyphenols, total flavonoids, caffeine, soluble sugars, theaflavins, and catechins, alongside an increase in free amino acids and theabrownin. The declining trend in catechins is further supported by the data on catechin monomers in Table 2.

Caffeine, a key alkaloid found in tea, is renowned for its bitter flavor and health-promoting properties [26], decreased by 22.48% after fermentation. The reduction in soluble sugars can be attributed to their consumption by *G. lucidum* for growth and metabolism. Similarly, Huang et al. [27] found a decrease in soluble sugars during early dark tea fermentation by *E. cristatum*, mainly due to microbial consumption. In dark teas, microbial fermentation significantly reduces tea polyphenols, catechins, and amino acids [28,29]. Conversely, *G. lucidum* fermentation significantly increases amino acid content, likely due to proteolytic enzyme activity and subsequent protein hydrolysis.

Green tea’s primary bioactive compounds are eight flavan-3-ol monomers or catechins. Table 2 details the quantification of key catechins: epigallocatechin gallate (EGCG), gallocatechin gallate (GCG), epicatechin gallate (ECG), catechin gallate (CG), epigallocatechin (EGC), gallocatechin (GC), epicatechin (EC), and catechin (C). In brewed green tea, catechins constitute 30–42% of the dry weight of soluble solids, with EGCG accounting for 50–80% of total catechins [30].

Table 2 shows a significant reduction in the eight catechin monomers after *G. lucidum* fermentation. Compared to CK, the contents of EGCG, GCG, ECG, CG, EGC, GC, EC, and C decreased by 99.68%, 99.77%, 98.44%, 98.61%, 99.86%, 99.94%, 92.71%, and 73.38%, respectively (*p* < 0.01) on day 12. The degradation showed a linear decrease over time, with the highest rate in the first 4 days and a slowdown for C, EC, EGC, GCG, and GC from days 8 to 12. Minimal catechin levels were also similar to black tea [31]. Fermentation reduces the bitterness and astringency of tea caused by decreasing catechin levels [32].

Many fungi, including *Chaetomium cupreum*, *Aspergillus niger*, *Penicillium commune*, and *Eurotium cristatum*, can degrade catechins [27,33,34]. Notably, *A. niger* (PSH) and *P. commune* (EH2) achieve degradation rates of 79.33% and 76.35%, respectively [34]. *C. cupreum* cleaves catechins into intermediates like catechol and pyruvate [33]. *E. cristatum*’s hydrolases break down ester-type catechins into gallic acid and non-esterified catechin monomers [35]. These findings highlight diverse fungi capable of catechin degradation and their intricate pathways. However, the degradation pathways and end products of catechins during *G. lucidum* fermentation in green tea remain undocumented.

Following fermentation with *G. lucidum*, theaflavins decreased by 2.73-fold, while theabrownins increased by 10.21-fold. The rise in theabrownins is associated with the transformation of tea polyphenols, catechins, theaflavins, and thearubigins. Phenolic compounds like catechins oxidize to form quinones, which polymerize into thearubigin and theaflavin. These interact with polysaccharides and other compounds to produce theabrownins. Quinones can also form theabrownin directly through oxidation and polymerization [27]. We suggest that *G. lucidum* enzymes convert catechins into theaflavin and thearubigin, which further oxidize into theabrownins, explaining the observed changes. This conversion alters tea color from greenish-yellow to reddish-brown and the taste from bitter to mellow, enhancing sensory qualities [36].

After fermenting green tea with *G. lucidum*, the changes in catechin, theaflavin, and theabrownins content mirror those in dark tea fermentation, suggesting universal biochemical changes across tea fermentation processes [37]. Our research shows similarities between *G. lucidum*-fermented tea and microbially fermented dark tea in chemical transformations. During fermentation, chemical components undergo degradation (e.g., EGCG into gallic acid), oxidation (catechins into theaflavins), condensation (theaflavins polymerizing into thearubigins and theabrownins), and structural modifications (glycosylation, hydroxylation, methylation) [38].

### 3.2. Analysis of Total Metabolites During the Fermentation

Metabolomic techniques are increasingly used in tea research for comprehensive constituent analysis. However, most methods rely on either targeted detection, limiting detectable substances, or non-targeted detection, which lacks sensitivity and precision, potentially leading to false positives [13,39]. In contrast, widely targeted metabolomics combines the strengths of both approaches, offering high throughput, high sensitivity, and broad coverage [13,40]. This study utilized widely targeted metabolomics to identify the most metabolites in the samples. A total of 2772 metabolites were detected using UPLC-MS/MS and GC-MS platforms (Table S1, total metabolites table). PCA analysis (Figure S1) revealed significant differences among the four groups, with fermented samples clustering more closely and distinctly compared to CK.

A heatmap was used to visualize the dynamic changes in metabolites across groups (Figure S2). Metabolites in the CK group showed higher relative content in the upper part of the heatmap (red dotted box) but were lower in the treatment groups. In contrast, metabolites in the lower part were more abundant in fermented tea than in the CK group. To assess the effect of fermentation, orthogonal partial least squares–discriminant analysis (OPLS-DA) was performed on 2772 metabolites for CK vs. Treat, CK vs. Treat-d4 vs. Treat-d12, and CK vs. Treat-d8 vs. Treat-d12. Figure S3a shows clear separation between the CK (left) and fermented samples (right), while Figure S3b,c show distinct differences among samples at different fermentation stages, with CK on the lower left, Treat-d12 in the upper middle, and Treat-d4 and Treat-d8 on the lower right.

### 3.3. Evolution of the Differential Metabolites During Fermentation

**Figure 1:**
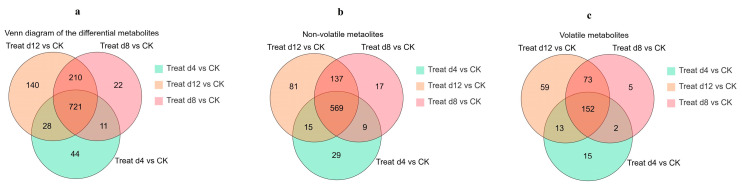

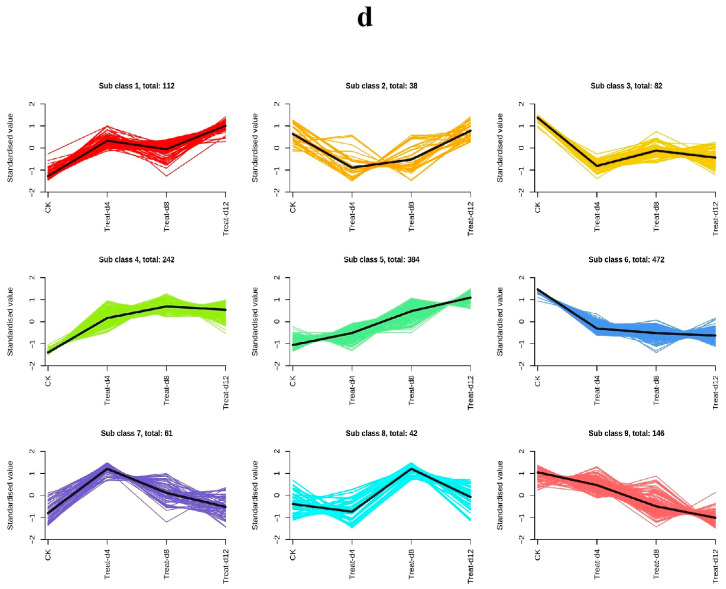
Composite changes in green tea infusion of different fermentation periods ((**a**) Venn diagram of all differential metabolites; (**b**) Venn diagram of differential non-volatile metabolites, GC-MS platform; (**c**) Venn diagram of differential volatile metabolites, UPLC-MS/MS platform; (**d**) k-means clustering algorithm analysis of metabolites. CK, control group; Treat-d4, the 4th-day fermentation group; Treat-d8, the 8th-day fermentation group; Treat-d12, the 12th-day fermentation group), *n* = 3.

Metabolites were screened based on VIP > 2 and fold change ≥2 or ≤0.5 [40]. Figure S4 shows that compared to the CK group, the Treat-d4, Treat-d8, Treat-d12, and Treat groups had 427, 510, 560, and 484 up-regulated substances, and 377, 454, 539, and 411 down-regulated substances, respectively. Figure 1 highlights both common and unique metabolites among the groups, with Treat-d12 vs. CK showing the most unique metabolites (81 non-volatile and 59 volatile). The three groups shared 721 differential metabolites. Nine distinct trends were observed (Figure 1d), with 738 metabolites elevated and 700 reduced in Treat groups compared to CK.

#### 3.3.1. Flavonoids

Figure S5 shows significant alterations in non-volatile compounds during fermentation, with increased nucleotides and amino acids, while flavonoids and phenolic acids decreased. The main flavonoids in tea, such as kaempferol, myricetin, quercetin, apigenin, and luteolin, have low bioavailability. In this study, 22 kaempferol, 10 myricetin, 16 quercetin, 6 apigenin, and 9 luteolin compounds significantly decreased (Figure S5a). Previous research suggests that glycosylation and methylation improve bioavailability [38]. Moreover, Cao et al. [41] found that microorganisms significantly modify polyphenol structures, increasing the bioavailability of these compounds.

We identified 82 glycosylated, 5 methylated, and 21 hydroxylated flavonoids, all decreasing after fermentation (Table S2, Table of overall differential metabolites). The 82 flavonoids were biotransformed into mono-, di-, and triglycosides: kaempferol-4′-O-(0.42-fold), kaempferol-3-O-(0.30-fold), apigenin-4′-O-(0.05-fold), quercetin-7-O-(0.34-fold), quercetin-4′-O-(0.31-fold), quercetin-3-O-(0.29-fold), luteolin-3′-O-(0.31-fold), luteolin-4′-O-(0.31-fold), luteolin-7-O-(0.31-fold), myricetindiglucoside (0.18-fold), kaempferol-3,7-O-diglucoside (0.18-fold), and chrysoeriol triglucoside (0.48-fold). Decreased glycosides may reduce tea astringency and bitterness [13].

One hydroxylated flavonoid, 2-hydroxy-2,3-dihydrogenistein, increased 18.82-fold, indicating microbial transformation into its di-hydroxylated form (Table S2). Flavonols and flavones, though present in low amounts, strongly influence astringency and bitterness. In this study, 63 flavonols and 37 flavones significantly decreased, leading to reduced bitterness. Similar results have been observed in dark tea, where flavonol and flavone levels drop by about 65% after fermentation, influencing taste [38,42].

#### 3.3.2. Amino Acids and Their Derivatives

The umami taste and aroma of tea are closely tied to its amino acid composition. Free amino acids, particularly theanine, glutamic acid, and aspartic acid, contribute about 70% of the umami flavor in green tea. Alanine and arginine add sweetness, while tryptophan and phenylalanine increase astringency and bitterness [38]. Fermentation did not alter the levels of these amino acids, indicating that *G. lucidum* fermentation does not affect the taste qualities they impart.

We observed a significant increase in 131 peptides and a decrease in 12 (Figure S5b). The free amino acid content in the day-12 fermented tea increased 2.56-fold to 0.238 g/mL compared to CK (Table 1). Tea protein hydrolysis produces smaller peptides rich in polar residues, enhancing hydrogen bonding and protein solubility. It is speculated that enzymes from *G. lucidum* may alter the size, polarity, and solubility of tea proteins. Additionally, the generated peptides may enhance antioxidant, antihypertensive, and antidiabetic properties [43].

#### 3.3.3. Phenolic Acids

Our research shows *G. lucidum*’s involvement in the consumption and biotransformation of phenolic acids. Dihydroferulic acid, 3-(3-hydroxyphenyl)-3-hydroxypropanoic acid, 2-phenylethanol, sibiricose A3, and caftaric acid increased by 10.18-fold, 15.96-fold, 16.57-fold, 17.75-fold, and 81.82-fold, respectively (Figure S5c). Meanwhile, major phenolic acids like gallic acid and caffeic acid decreased after fermentation. Some caffeic acid derivatives, such as 1-O-caffeoyl-β-D-xylose and cynarin, were increased. Gallic acid, crucial for the umami taste in tea [44], and its 34 derivatives decreased, while galloyl xylosyl gallic acid-3-O-β-D-glucoside increased. The detection of methylated and hydroxylated phenolic acids suggests microbially mediated structural changes.

#### 3.3.4. Nucleotides and Derivatives

A nucleotide consists of a nucleoside (a nucleobase and a ribose or deoxyribose sugar) and one or more phosphate groups. Mushrooms, including *G. lucidum*, are rich in nucleosides [45]. Peng et al. [46] identified adenosine, guanosine, inosine, cytidine, thymidine, uridine, and 2′-deoxyadenosine in *G. lucidum*, and similar compounds were detected in this study. After fermentation, adenosine, guanosine, cytidine, thymidine, uridine, and 2′-deoxyadenosine increased by 10.18-fold to 90.26-fold (Figure S5d). Other nucleosides, including 2′-deoxycytidine, 2′-deoxyguanosine, and 2′-deoxyinosine, showed significant increases, as also identified by Chen et al. [47] in *G. lucidum*. Additionally, guanosine 5′-monophosphate, a key nucleotide in RNA and cell metabolism [48], was detected in the fermented green tea infusion.

Nucleosides and nucleotides regulate immune function, fatty acid metabolism, and iron absorption and aid gastrointestinal recovery [49,50]. Nucleotide-enriched foods, including those produced by *G. lucidum* fermentation, may support recovery during illness or immune dysfunction. Further research is needed to verify the biological functions of these nucleotides. The umami flavor in mushrooms is largely due to 5′-nucleotides like 5′-guanosine monophosphate and 5′-inosine monophosphate [45]. In this study, 5′-guanosine monophosphate and 5′-uridine monophosphate increased by 7.24-fold and 13.05-fold, contributing to the enhanced umami flavor of *G. lucidum*-fermented tea.

### 3.4. Changes in the Volatile Metabolites During Fermentation

**Figure 2:**
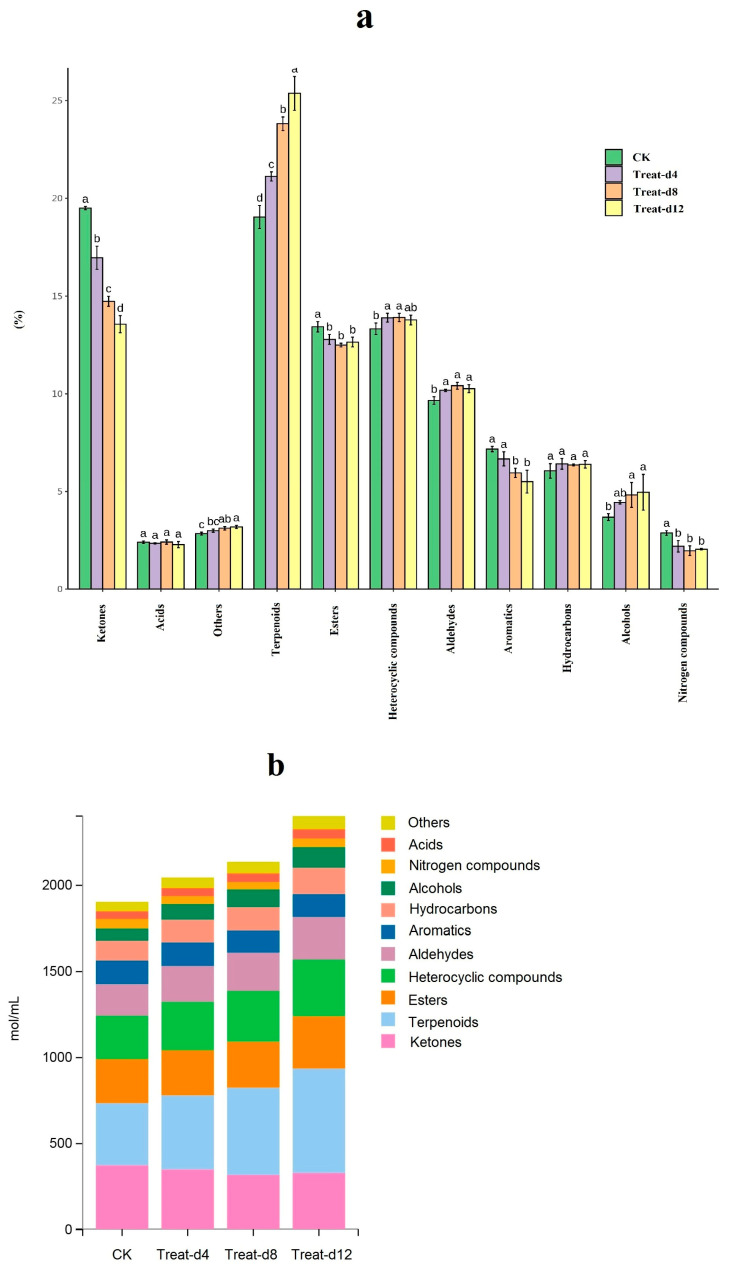
Changes in the volatile metabolites during fermentation ((**a**) proportions of different volatile metabolite types in green tea infusion; (**b**) total content of volatile metabolites during the different periods. CK, control group; Treat-d4, the 4th-day fermentation group; Treat-d8, the 8th-day fermentation group; Treat-d12, the 12th-day fermentation group), *n* = 3. The superscript letters (a, b, c, d) indicate statistically significant differences between groups.

In this study, fermentation with *G. lucidum* significantly increased the levels of terpenoids, aldehydes, and alcohols, while reducing ketones, esters, aromatics, and nitrogen compounds. The total volatile metabolites increased after fermentation (Figure 2a,b). Terpenoids, known for their low sensory thresholds and pleasant fragrances, are crucial to tea’s aroma. Compounds like linalool, its oxides, nerolidol, and others were identified via GC-MS [51] and are common in teas [52].

During Fu brick and Pu-erh tea processing, microbial glycosidases break down glycosides, forming linalool and its oxides, which enhance the sweet and floral aroma of dark tea [27,53]. In this study, linalool increased from 1.60 mol/mL initially to 3.46 mol/mL by the end of fermentation. Trans-linalool oxide (furanoid) peaked at 0.78 mol/mL after 12 days. Increases in phellandrene and stable levels of citronellal, α-ionone, limonene, and α-terpineol were observed post-fermentation.

Aldehydes and ketones from sugar thermal degradation can react with free amino acids and amines to form Schiff bases, leading to furfural derivatives. Sugars may also dehydrate, condense, and polymerize, changing the infusion color and enhancing its aroma [54]. Post-fermentation, 11 aldehydes, 12 ketones, and furfural derivatives increased (Table S2). Using Saccharomyces cerevisiae in green tea fermentation raises levels of alcohols, esters, and acids, enhancing floral and fruity aromas [55]. Notably, *G. lucidum* fermentation increased geraniol by 1.80-fold and 2-phenylethanol by 1.66-fold, altering tea’s flavor profile.

### 3.5. Taste and Aroma Characteristics of Green Tea Infusion

**Figure 3:**
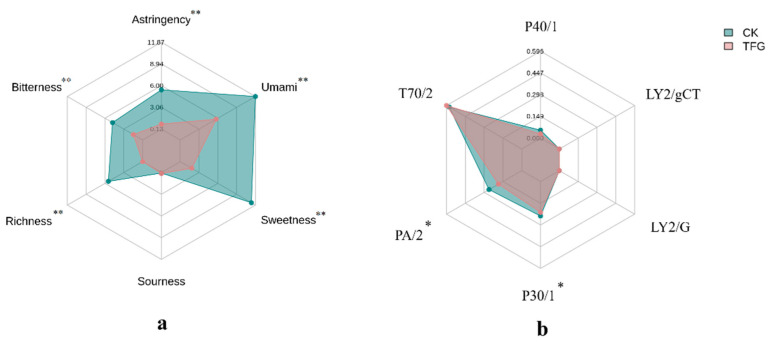
Radar plot ((**a**) taste profiles based on the responses of E-tongue sensors; (**b**) sensor response values detected by the E-nose), *n* = 3. The symbols * and ** in the figure denote statistical significance at *p* < 0.05 and *p* < 0.01, respectively.

As shown in Table 3, fermentation causes fundamental changes in the color, aroma, and taste of green tea. The aroma shifts from slightly grassy to woody and sweet, while the taste transitions from fresh and slightly astringent to mellow and floral. E-tongue evaluation of TFG (Figure 3a) revealed a significant decrease (*p* < 0.01) in bitterness, astringency, umami, sweetness, and richness after fermentation.

The E-nose measured odor signals from tea samples, with the radar chart (Figure 3b) showing significantly decreased response values in TFG for sensors PA/2 and P30/1, compared to CK (*p* < 0.05), while T70/2 slightly increased. The response values for sensors LY2/gCT and LY2/G were near zero. Analysis revealed that sensors detecting aromatic compounds, flammable gaseous organic compounds (P30/1; e.g., hydrocarbons), and pungent gaseous compounds (PA/2; e.g., amines, ethanol, ammonia) showed a significant reduction in TFG compared to CK. These results suggest that fermentation reduces pungent and flammable gaseous compounds in green tea.

### 3.6. Analysis of Key Aroma-Active Compounds in TFG

Aroma-active compounds in tea were assessed by analyzing their odor thresholds and concentrations, using relative odor activity values (rOAVs) for evaluation. Volatiles with rOAV >1 are considered key flavor constituents, with 35 volatiles meeting this criterion (Table S3, rOAV for volatile aroma compounds in green tea infusion, Figure S6) [56].

The degradation of threonine produces 5-ethyl-3-hydroxy-4-methyl-2(5H)-furanone, imparting a sweet, maple, caramel aroma [57]. It exhibited the highest rOAV in the green tea infusion. In the CK group, 1-octen-3-one had the second-highest rOAV (833.71), while in the treated group, benzenemethanethiol was second (962.00), contributing a smoky aroma similar to boxwood and certain wines [58]. During fermentation, the rOAV of 1-octen-3-one decreased, indicating reduced mushroom flavor post-*G. lucidum* fermentation.

Key aroma compounds, including benzenemethanethiol, (Z,Z)-3,6-nonadienal, methyl benzoate, geraniol, naphthalene, linalool, benzeneacetaldehyde, and 3-methoxy-2,5-dimethylpyrazine, peaked in the final fermentation stage, enhancing the tea’s aroma. Benzeneacetaldehyde, with a rose-like scent, was crucial in black tea aroma [59]. Extracellular enzymes from fungal fermentation, especially those from *G. lucidum*, catalyze monoterpene alcohol production, such as linalool, reaching peak rOAV between 8 and 12 days [53]. Geraniol, noted for its rose-like fragrance, is vital in black and white tea aroma formation [60,61]. In Benshan tea, geraniol, and benzeneacetaldehyde contribute to its floral and fruity characteristics [62].

The influence of *G. lucidum* fermentation shifted tea aroma from green to woody, floral, sweet, and fruity. Differential metabolite analysis (Figure S7, Table S3) shows a strong correlation between the woody aroma and compounds like β-pinene, α-phellandrene, and others. Floral aromas were linked to linalool oxides, α-bisabolol, and 10-epi-γ-eudesmol, while the sweet aroma was associated with various terpenes, such as trans-β-ocimene and (+)-3-carene. The woody and herbal notes were connected to α-phellandrene and spirocyclic compounds, with floral and fruity aromas tied to geraniol and citronellyl tiglate.

### 3.7. Analysis of In Vitro Antioxidant and Lipid-Lowering Activities of TFG and Associated Metabolites

#### 3.7.1. Changes in Antioxidant and Lipid-Lowering Activities of Green Tea Infusion After Fermentation

This study assessed antioxidant activities using DPPH and ABTS·+ free radical scavenging assays (Table 4). Fermented green tea infusion significantly reduced both DPPH and ABTS·+ levels, with DPPH reaching its minimum on the 8th day and ABTS·+ on the 4th day, after which values gradually increased. Fermentation reduced DPPH and ABTS values of the green tea infusion by 53.49% and 61.01%, respectively. The main antioxidant components, gallic acid and catechins (especially EGCG and ECG) [63], decreased by 49.27% and 96.34%. Thus, antioxidant activities significantly declined after fermentation, corresponding with changes in gallic acid and catechin levels.

As shown in Table 4, the binding capacity of TFG to bile salts (sodium glycinate and sodium taurocholate) was evaluated to explore potential lipid-lowering effects. TFG’s binding capacity to bile salts significantly increased with fermentation. Sodium glycinate demonstrated a stronger binding affinity than sodium taurocholate for TFG. The maximum binding rates for Treat-d12 to sodium glycinate and sodium taurocholate were 51.58% and 39.90% (*p* < 0.01), respectively. We speculate that theabrownins are the main contributors to lipid-lowering properties in TFG. Kuo et al. [64] showed that dark tea, with a higher theabrownins level, induces greater hypolipidemic effects than other teas. Subsequent studies [65,66] support the role of theabrownins in reducing cholesterol levels and preventing hyperlipidemia in obesity models. Xiao et al. [67] highlighted the lipid-lowering activity of specific theabrownins fractions (10–30 kDa) in HFD-fed zebrafish.

#### 3.7.2. Association Between Differential Metabolites and Antioxidant and Lipid-Lowering Properties

**Figure 4:**
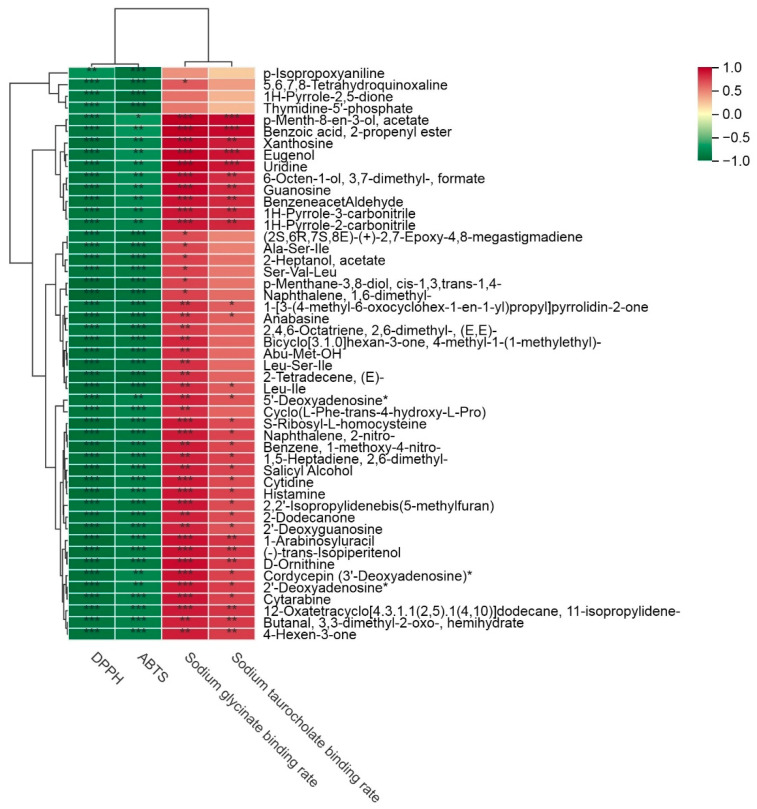
The correlation results between the differential metabolites (VIP ≥ 1.50) and antioxidant and lipid-lowering activities, *n* = 3. The symbols *, **, and *** in the figure denote levels of statistical significance, with more stars indicating greater significance (*: *p* < 0.05, **: *p* < 0.01, ***: *p* < 0.001).

Pearson correlation analysis was conducted between significantly altered metabolites (VIP ≥ 1.50) during fermentation and antioxidant and lipid-lowering activities (Figure 4). In this study, lipid-lowering effects showed strong positive correlations with p-menth-8-en-3-ol acetate, benzoic acid 2-propenyl ester, xanthosine, eugenol, uridine, 6-octen-1-ol 3,7-dimethyl-formate, guanosine, benzeneacetaldehyde, 1H-pyrrole-3-carbonitrile, and 1H-pyrrole-2-carbonitrile. The top 50 differential metabolites had a significant negative correlation with the antioxidant activities of TFG.

## 4. Conclusions

Based on 2772 identified metabolites, this study elucidated biomolecular alterations in *G. lucidum*-fermented green tea infusion as a functional food beverage. Fermentation significantly reshaped TFG’s sensory profile and bioactivities: Bitterness decreased due to reductions in flavonoids (−87.10%), catechins (−97.02%), and caffeine (−22.48%); the mellow umami taste increased via elevated theabrownins (+10.21-fold) and 5′-nucleotides (+7.24-fold to 13.05-fold); the aroma shifted from grassy to woody/sweet notes, with 35 key aroma-active compounds (OAV > 1) identified; antioxidant capacity diminished (−53.49% to 61.01%) due to reduced gallic acid/catechins, whereas lipid-lowering potential enhanced by 92.24–116.26% through enzymatic oxidation-induced theabrownins accumulation. These findings provide crucial insights into how *G. lucidum* fermentation transforms green tea into a novel beverage with enhanced lipid-lowering functionality and distinct sensory properties. The scale of metabolite alteration underscores targeted metabolomics’ value in decoding functional food fermentation.

## Figures and Tables

**Table 1:** Determination of the major chemical components of TFG.

Item	CK	TFG
Tea polyphenol, g/100 mL	3.06 ± 0.83	1.01 ± 0.12
Total flavonoids, g/100 mL	0.486 ± 0.091	0.0627 ± 0.006
Caffeine, mg/L	905.87 ± 26.24	702.19 ± 49.08
Soluble sugar, g/100 mL	0.36 ± 0.07	0.21 ± 0.03
Free amino acid, g/100 mL	0.093 ± 0.005	0.238 ± 0.028
Theaflavins, mg/L	3183.07 ± 180.85	1165.85 ± 33.83
Theabrownins, mg/100 mL	1.44 ± 0.08	14.70 ± 1.50

CK, control group; TFG, green tea infusion fermented by *Ganoderma lucidum*. Values were the results of analysis conducted in duplicate.

**Table 2:** The catechin monomers in dried green tea infusion (μg/g).

Constituent	CK	Treat-d4	Treat-d8	Treat-d12 (TFG)	SEM	*p*-Value	Linear
Epigallocatechin gallate, EGCG	36,077.55 ± 4763.05 ^a^	802.40 ± 407.13 ^b^	306.83 ± 76.34 ^b^	113.07 ± 5.07 ^b^	1656.12	<0.01	<0.01
Gallocatechin gallate, GCG	21,914.94 ± 988.83 ^a^	363.05 ± 137.75 ^b^	82.04 ± 6.32 ^b^	51.74 ± 5.24 ^b^	337.31	<0.01	<0.01
Epicatechin gallate, ECG	12,763.01 ± 1409.09 ^a^	845.99 ± 537.58 ^b^	518.93 ± 6.07 ^b^	198.74 ± 12.88 ^b^	586.33	<0.01	<0.01
Catechin gallate, CG	14,008.53 ± 173.37 ^a^	961.45 ± 623.34 ^b^	543.04 ± 27.76 ^b^	196.56 ± 10.81 ^b^	247.69	<0.01	<0.01
Epigallocatechin, EGC	35,537.91 ± 2319.60 ^a^	102.07 ± 68.12 ^b^	43.55 ± 34.40 ^b^	48.34 ± 13.11 ^b^	831.66	<0.01	<0.01
Gallocatechin, GC	1598.29 ± 108.90 ^a^	4.50 ± 1.01 ^b^	0.40 ± 0.19 ^b^	0.94 ± 0.76 ^b^	38.70	<0.01	<0.01
Epicatechin, EC	11,937.85 ± 585.05 ^a^	2886.10 ± 176.90 ^b^	946.55 ± 151.08 ^c^	870.15 ± 15.64 ^c^	199.26	<0.01	<0.01
Catechin, C	10,602.68 ± 546.43 ^a^	5185.98 ± 1046.12 ^b^	3301.63 ± 403.64 ^c^	2821.21 ± 523.60 ^c^	434.34	<0.01	<0.01
Total	144,440.76 ± 2303.22 ^a^	11,151.55 ± 2728.33 ^b^	5742.97 ± 576.62 ^c^	4300.75 ± 493.52 ^c^	1308.23	<0.01	<0.01

CK, control group; Treat-d4, the 4th-day fermentation group; Treat-d8, the 8th-day fermentation group; Treat-d12 (TFG), the 12th-day fermentation group, *n* = 3. The superscript letters (a, b, c) indicate statistically significant differences between groups.

**Table 3:** Sensory evaluation of TFG.

Item	Liquor Color	Aroma	Taste
CK	yellowish-green, bright	slightly grassy	fresh, slightly astringent
TFG	reddish-brown, bright	woody, sweet	mellow, floral

CK, control group; TFG, green tea infusion fermented by *Ganoderma lucidum*.

**Table 4:** Changes in antioxidant activities of green tea infusions during fermentation.

Item	CK	Treat-d4	Treat-d8	Treat-d12 (TFG)	SEM	*p*-Value
DPPH (μmol/mL)	1434.85 ± 1.80 ^a^	820.49 ± 66.99 ^b^	630.33 ± 24.06 ^b^	667.31 ± 133.06 ^b^	57.48	<0.001
ABTS (μmol/mL)	37.70 ± 3.23 ^a^	9.53 ± 0.38 ^b^	11.61 ± 1.52 ^b^	14.70 ± 3.79 ^b^	2.05	<0.001
Sodium glycinate binding rate (%)	17.92 ± 1.82 ^b^	29.94 ± 1.19 ^c^	34.45 ± 4.27 ^b^	51.58 ± 1.17 ^a^	1.20	<0.001
Sodium taurocholate binding rate (%)	18.45 ± 0.70 ^c^	21.33 ± 0.44 ^c^	25.06 ± 1.50 ^b^	39.90 ± 2.36 ^a^	0.88	<0.001

CK, control group; Treat-d4, the 4th-day fermentation group; Treat-d8, the 8th-day fermentation group; Treat-d12 (TFG), the 12th-day fermentation group, *n* = 3. The superscript letters (a, b, c) indicate statistically significant differences between groups.

## Data Availability

The original contributions presented in this study are included in the article/Supplementary Materials. Further inquiries can be directed to the corresponding author.

## Supplementary Materials

The following supporting information can be downloaded at: https://www.mdpi.com/article/10.3390/foods14162855/s1. Figure S1: Principal component analysis plot; Figure S2: Hierarchical cluster analysis plot; Figure S3: Plot of OPLS-DA scores (a, CK vs. Treat; b, CK vs. Treat-d4 vs. Treat-d12; c, CK vs. Treat -d8 vs. Treat-d12.); Figure S4: Volcano plot of the differential metabolites presents in green tea infusion (a, Treat-d4 vs. CK; b, Treat-d8 vs. CK; c, Treat-d12 vs. CK; d, Treat vs. CK.); Figure S5: Heat map of the levels of the differential non-volatile metabolites during fermentation (5a, flavonoids; 5b, amino acids and derivatives; 5c, phenolic acids; 5d, nucleotides and derivatives); Figure S6: Scatter plot of relative odor activity values; Figure S7: Aroma radargram of fermented tea; Table S1: Total metabolites table; Table S2: Table of overall differential metabolites; Table S3, rOAV for volatile aroma compounds in green tea infusion.

## Abbreviations

The following abbreviations are used in this manuscript:

ABTS·^+^	2,2′-Azino-bis(3-ethylbenzothiazoline-6-sulfonic acid)
DPPH	2,2-Diphenyl-1-picrylhydrazyl
GC–MS/MS	gas chromatography–tandem mass spectrometry
rOAVs	relative odor activity values
TFG	green tea infusions fermented by *G. lucidum*
UPLC–MS/MS	ultra-performance liquid chromatography–tandem mass spectrometry
VOCs	volatile organic compounds
