# Supplementary figures and images for: Widely Targeted Metabolomics Decodes Metabolic Remodeling and Functional Shifts in Ganoderma lucidum-Fermented Green Tea Infusion
Source: Foods. 2025 Aug 18;14(16):2855. doi: 10.3390/foods14162855 (PMC12385791; doi:10.3390/foods14162855)

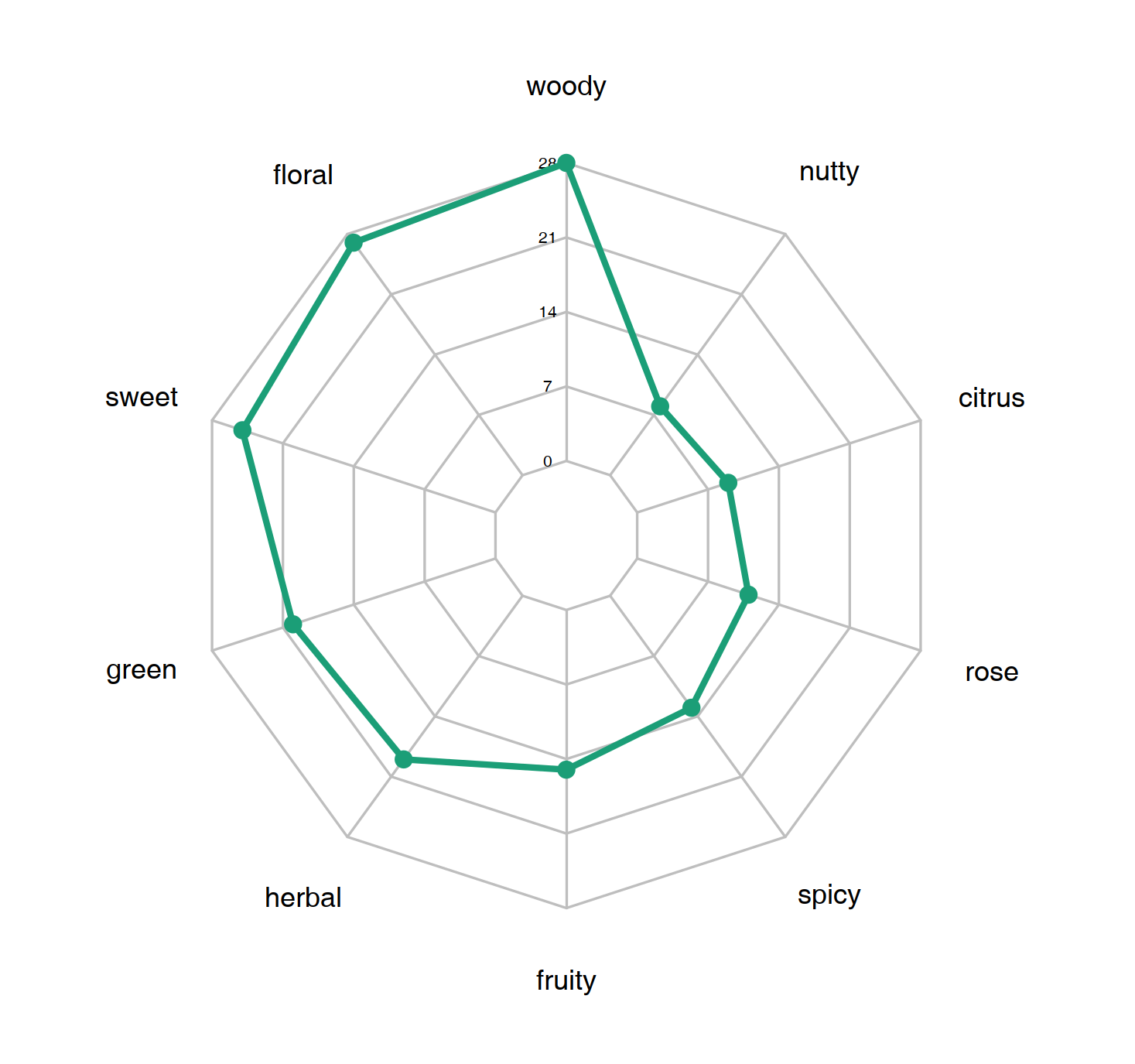

Supplement: Supplementary file 1 [file foods-14-02855-s001.zip › Figure_S7.png]

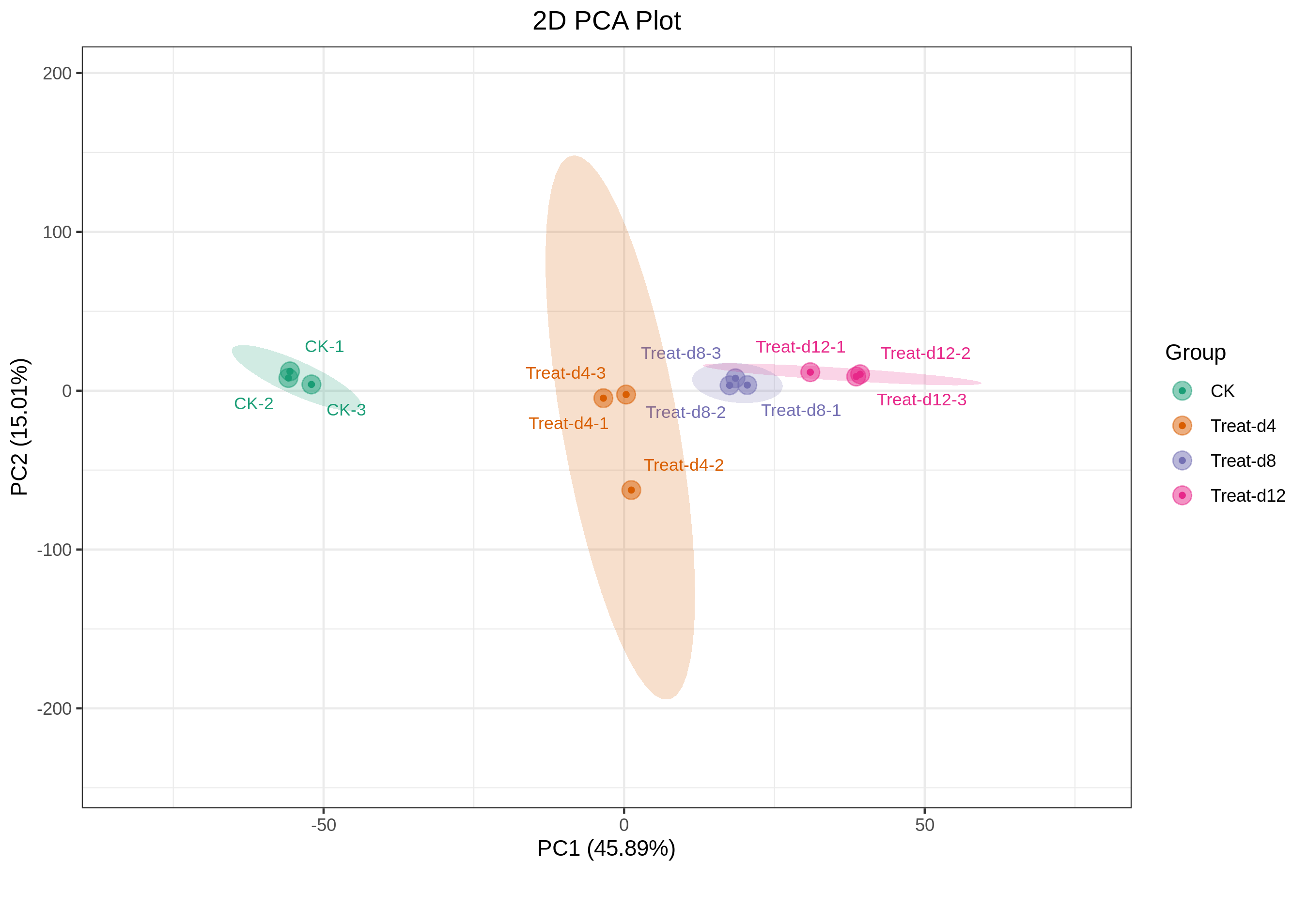

Supplement: Supplementary file 1 [file foods-14-02855-s001.zip › Figure_S1.png]

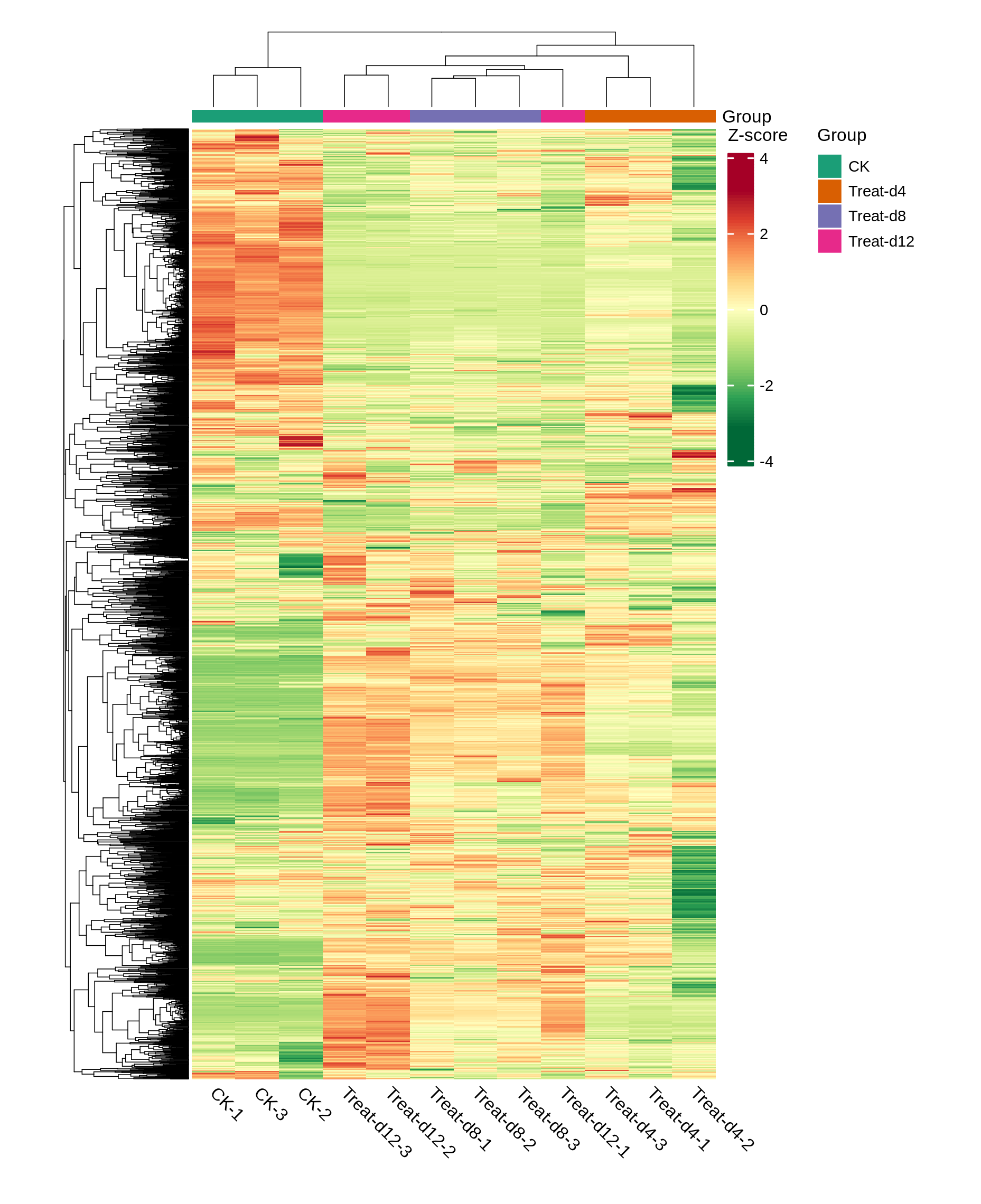

Supplement: Supplementary file 1 [file foods-14-02855-s001.zip › Figure_S2.png]

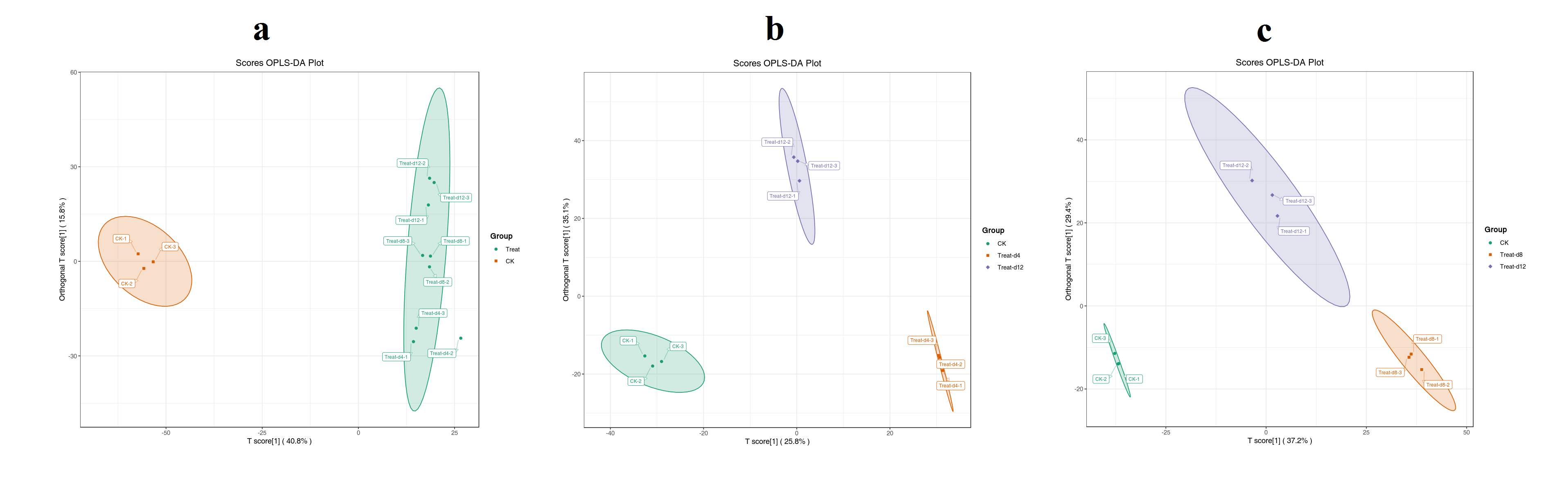

Supplement: Supplementary file 1 [file foods-14-02855-s001.zip › Figure_S3.png]

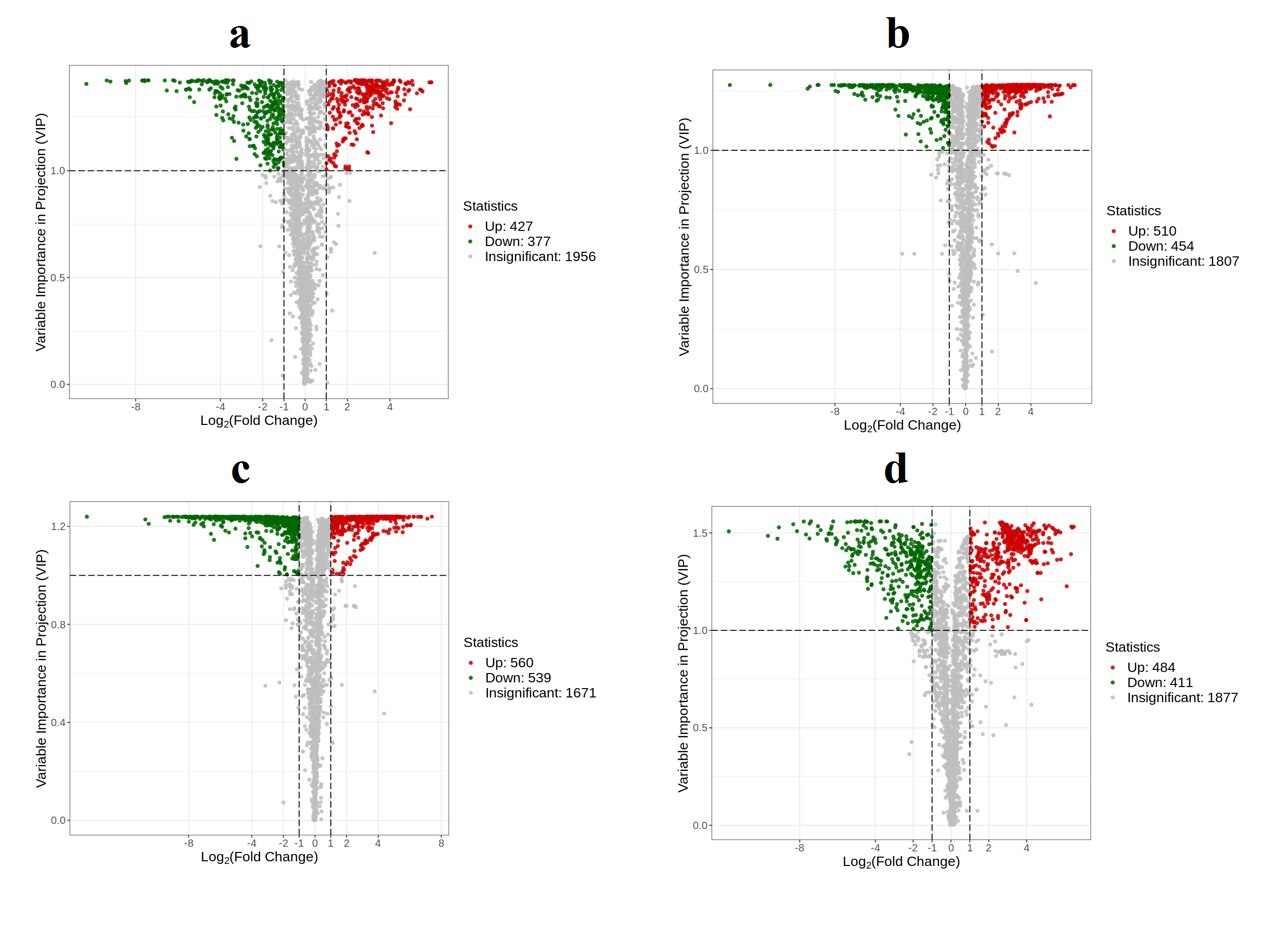

Supplement: Supplementary file 1 [file foods-14-02855-s001.zip › Figure_S4.png]

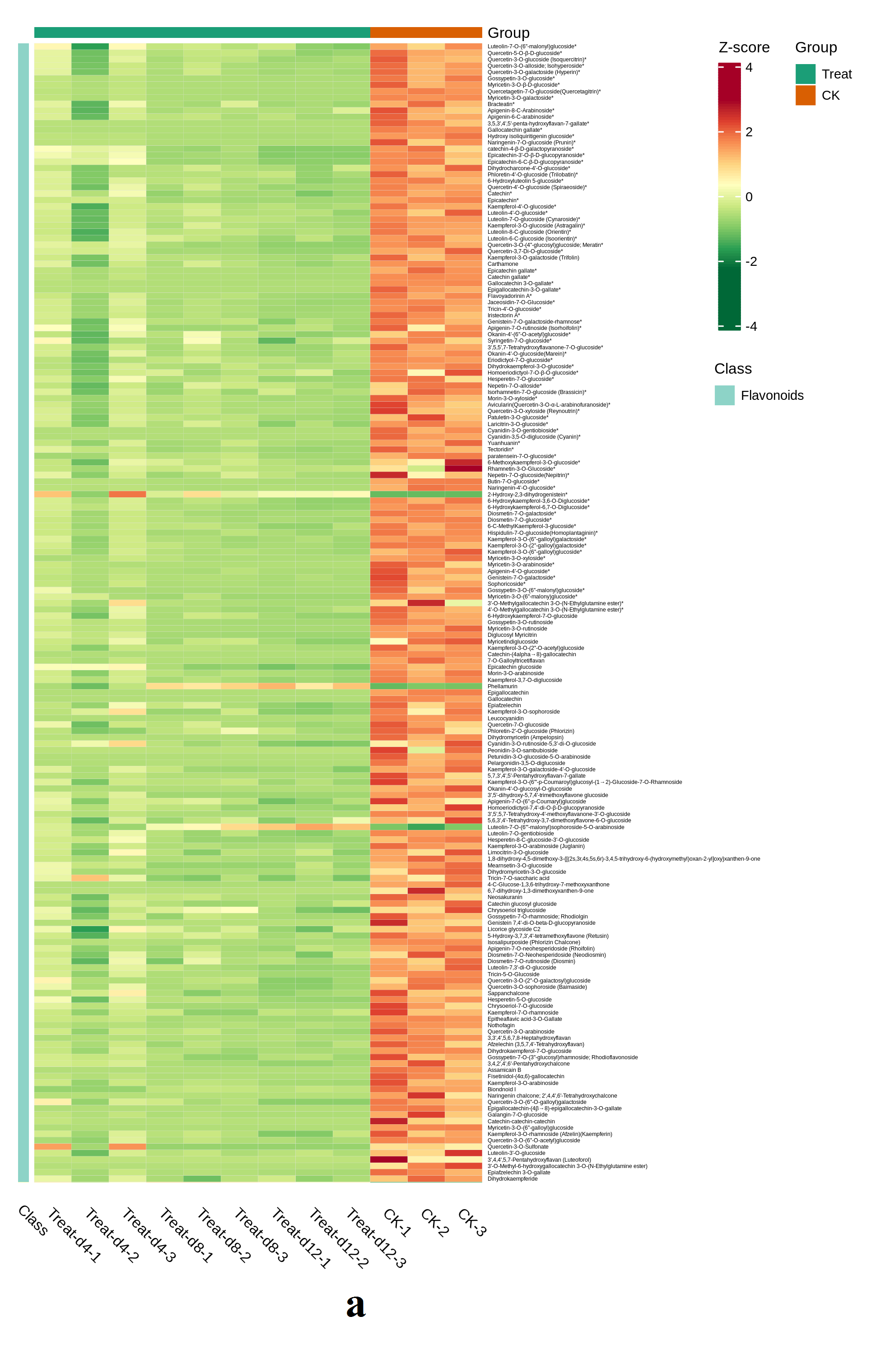

Supplement: Supplementary file 1 [file foods-14-02855-s001.zip › Figure_S5a.png]

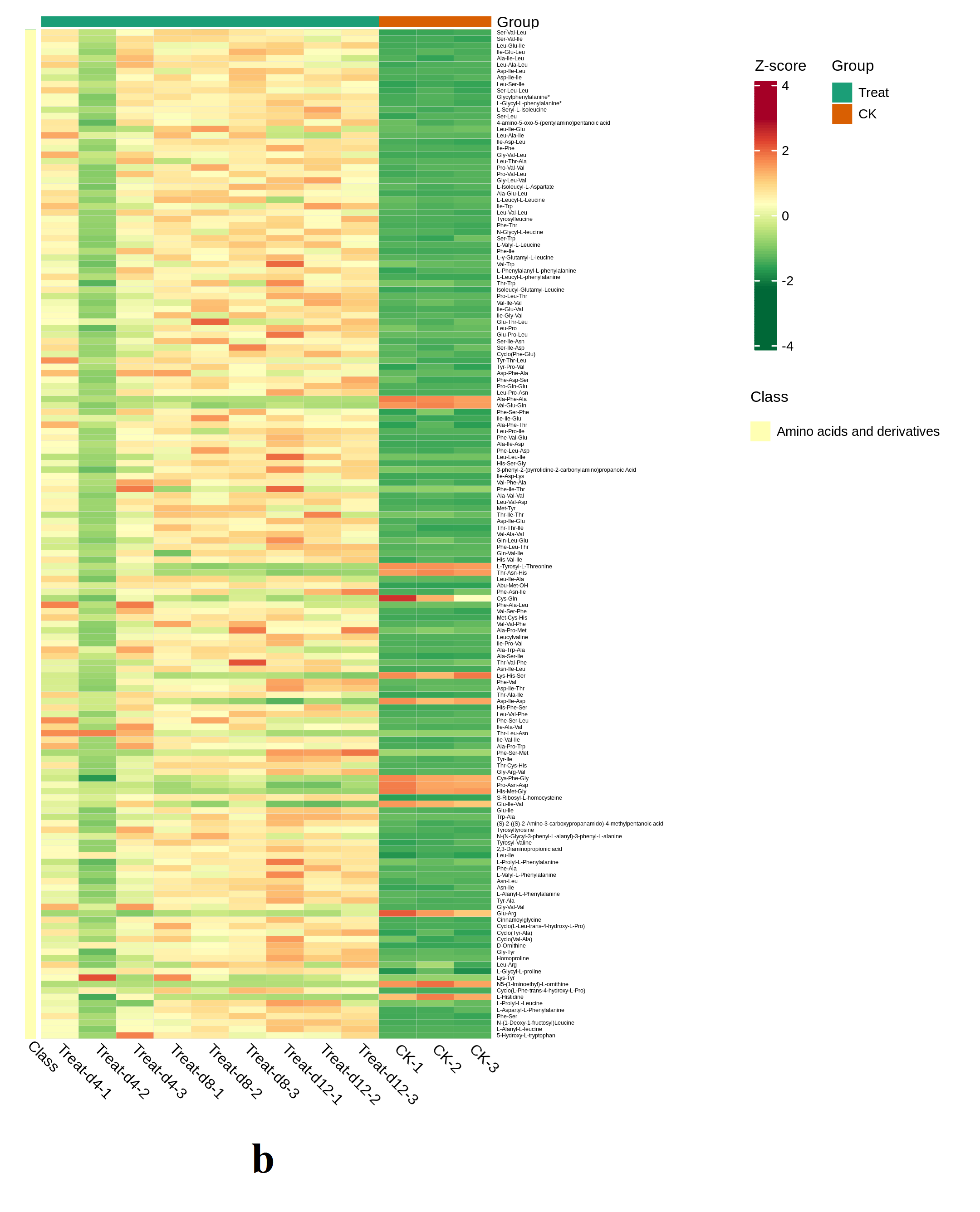

Supplement: Supplementary file 1 [file foods-14-02855-s001.zip › Figure_S5b.png]

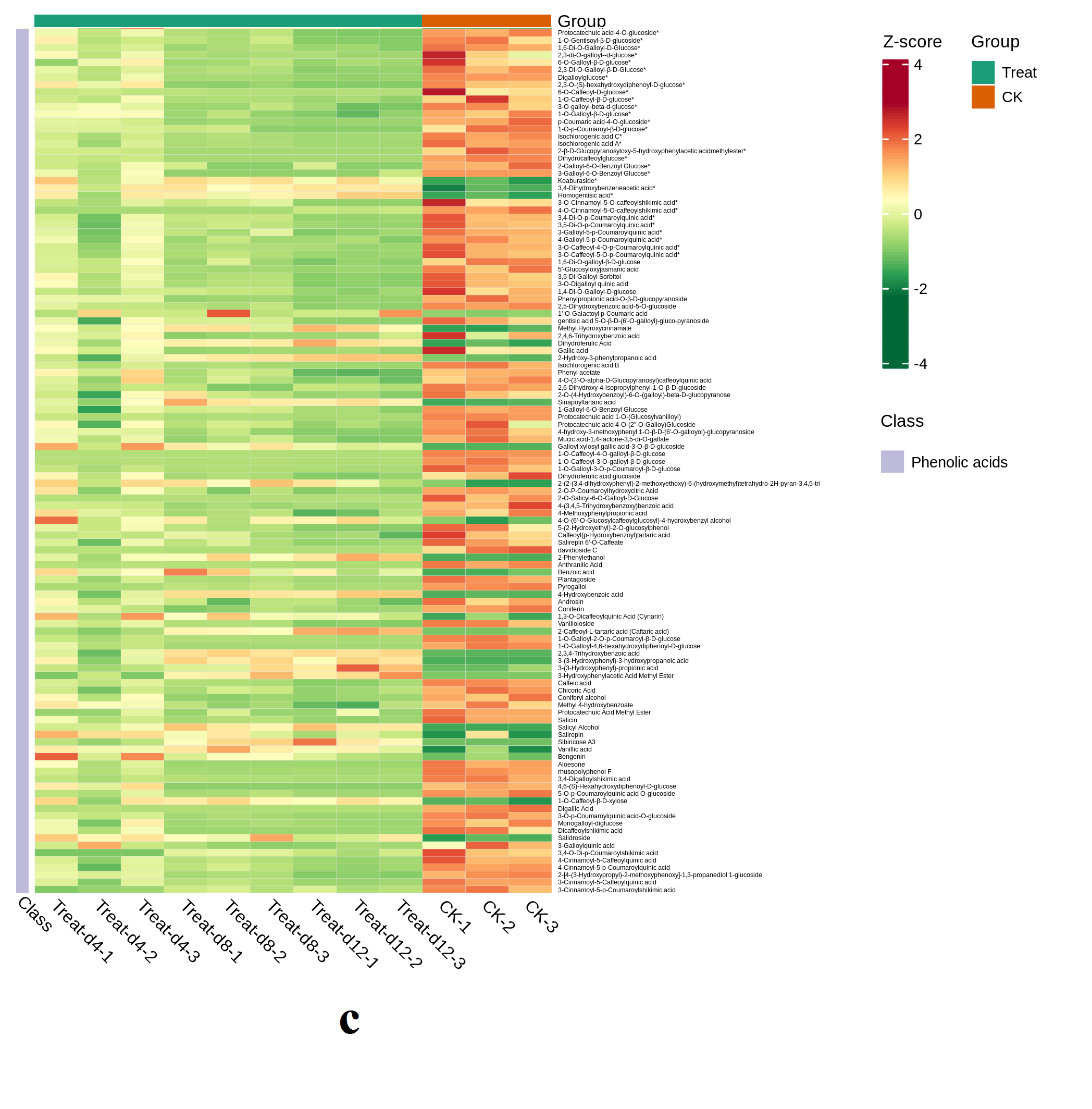

Supplement: Supplementary file 1 [file foods-14-02855-s001.zip › Figure_S5c.png]

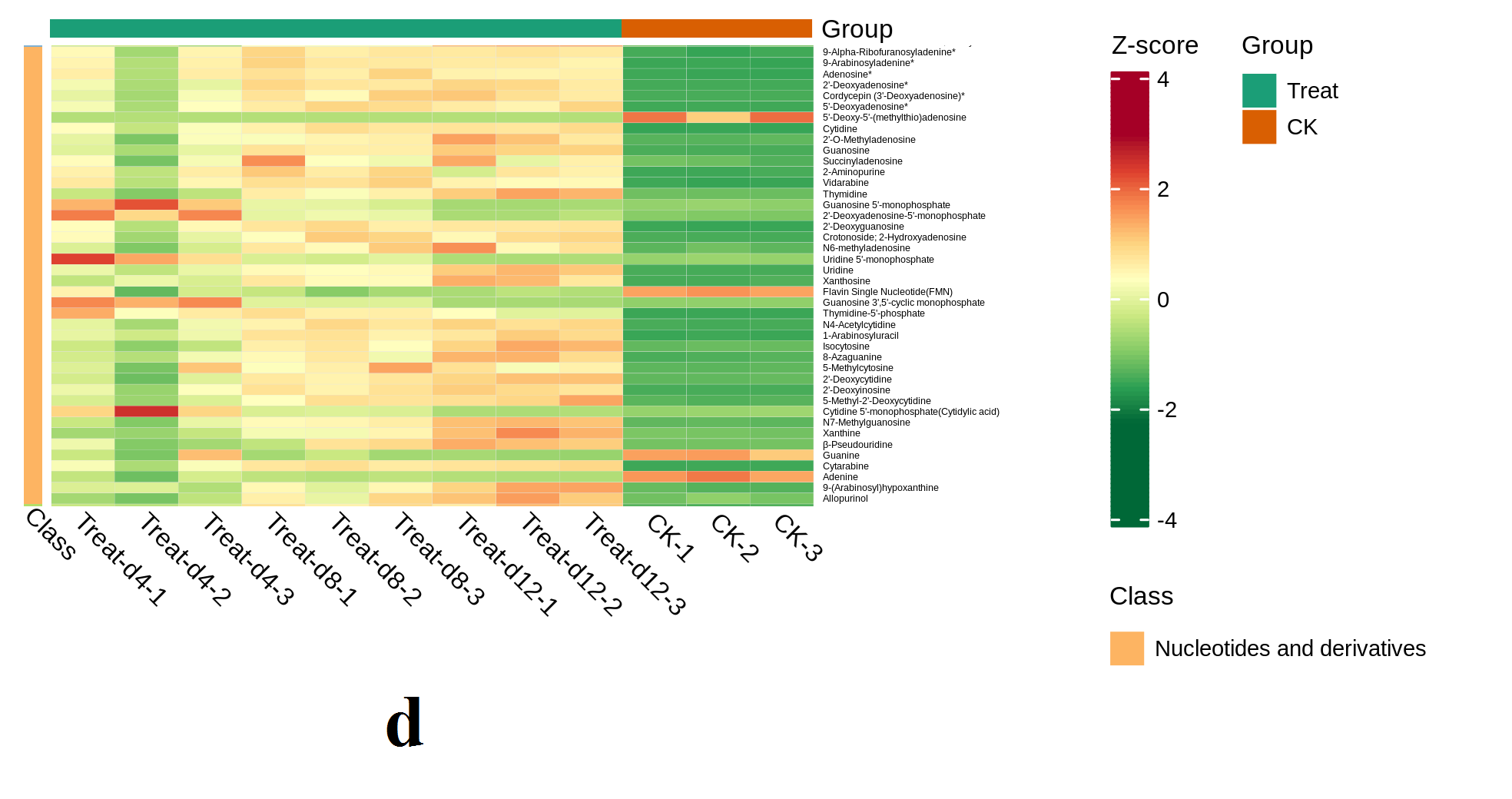

Supplement: Supplementary file 1 [file foods-14-02855-s001.zip › Figure_S5d.png]

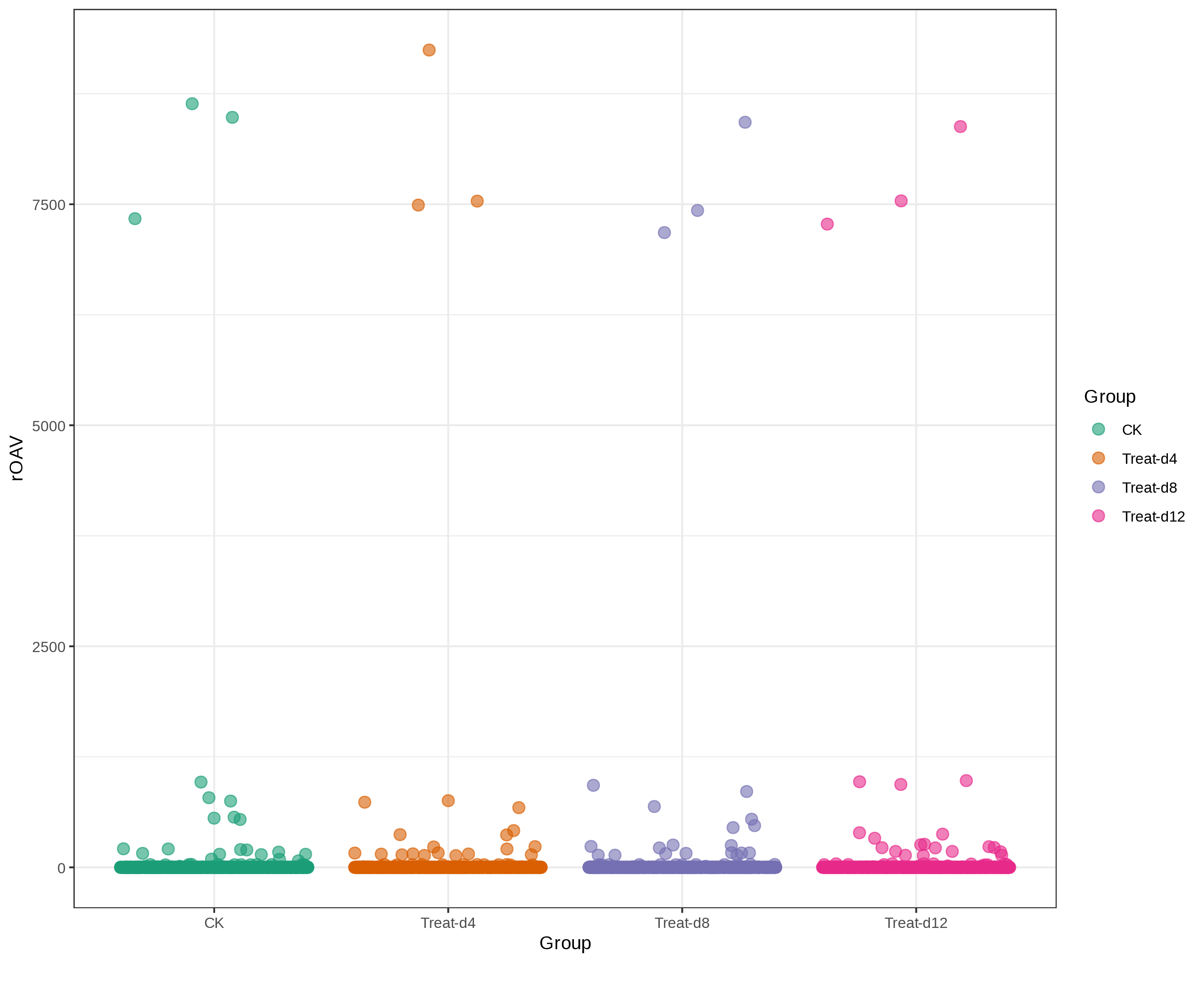

Supplement: Supplementary file 1 [file foods-14-02855-s001.zip › Figure_S6.png]
